# Rationale for launching a new journa

**DOI:** 10.1590/S1980-57642008DN10100001

**Published:** 2007

**Authors:** Ricardo Nitrini

**Affiliations:** Editor-in-Chief

**Keywords:** anatomy, vascular dementia, cognitive impairment, cholinergic fibers

## Abstract

Vascular cognitive impairment/vascular dementia have been the subject of a large number of studies, due to their high prevalence and broad preventive and compensatory therapeutic potential. The knowledge of the cerebral anatomy correlated to the vascular territories of irrigation enables understanding of clinical manifestations, as well as classification into the several types of syndromic presentations. The central cholinergic system exercises important neuromodulatory functions on cerebral circuits related to cognitive and behavioral integration, as well as on vasomotor control related to cerebral blood flow adjustments. The acquisition of data on the anatomy of the cholinergic pathways, including the localization of the nuclei of the basal prosencephalon and the routes of their projections, established an important milestone. The knowledge of the vascular distribution and of the trajectories of the cholinergic pathways allows identification of the strategic points where a vascular lesion can cause interruption. The ensuing denervation leads to cholinergic hypofunction in the involved territories. This information proves important to better evaluate the sites of vascular lesions, emphasizing their strategic localizations in relation to the cholinergic pathways, and offering more robust foundations for treatment aiming at enhancing cholinergic activity.

## Abstract (pt)

Comprometimento cognitivo vascular/demência vascular vem sendo objeto de numerosos estudos, levando em conta sua alta prevalência e as amplas possibilidades terapêuticas preventivas e compensatórias. O conhecimento da anatomia cerebral correlacionado ao dos territórios vasculares de irrigação permite a compreensão das manifestações clínicas, assim como a classificação dos diversos tipos de apresentações sindrômicas. O sistema colinérgico central exerce funções neuromoduladoras importantes dos circuitos relacionados à integração cognitiva e comportamental, além do controle vasomotor relacionado aos ajustes do fluxo sanguíneo cerebral. A obtenção de dados sobre a anatomia das vias colinérgicas, incluindo a localização dos núcleos do prosencéfalo basal e os trajetos das suas projeções, estabeleceu um marco importante. O conhecimento da distribuição vascular e do percurso das vias colinérgicas permite identificar pontos estratégicos onde a lesão vascular pode causar sua interrupção. A desnervação que se segue causa hipofunção colinérgica dos territórios acometidos. Essas informações são importantes para melhor avaliar os locais das lesões vasculares, enfatizando suas localizações estratégicas em relação às vias colinérgicas, oferecendo, desse modo, bases mais sólidas para o tratamento que visa aumentar a atividade colinérgica.

In the last two decades there has been a considerable increase in the Brazilian scientific production in many areas of knowledge, including the two closely related fields that constitute the core of **Dementia & Neuropsychologia**, a new scientific periodical.

It is important to present the other main reasons for launching this new journal in Brazil:

1. There is no journal specifically dedicated to these areas in the country, especially for research on dementia;
2. Versions to Brazilian-Portuguese or adaptations of internationally utilized tests, scales or questionnaires and studies focusing on peculiarities of the language problems of Portuguese-speaking patients may prove very difficult to publish in an international journal or in the national general journals of neurology, psychology or psychiatry;
3. When papers are not published in high level international journals, but in less well known international indexed journals, the paper often ends up virtually unknown by the national community;
4. Maybe these and other reasons have contributed to the low number of Brazilian studies that progress from poster form to full papers;
5. Last but not least, selected reviewers may improve the quality of the manuscripts, providing suggestions on how to adapt the scientific writing, statistical analyses and proper citation of the literature.

Of course, it will be difficult for a not yet indexed journal to attract good manuscripts. As **Dementia & Neuropsychologia** was approved as the official journal of the Cognitive Neurology and Ageing Department of the Brazilian Academy of Neurology and is receiving support from the most important groups related to the dementia and neuropsychological fields in Brazil, with an editorial board including members from most regions of Brazil, Latin America and abroad, it probably will receive manuscripts within the first year. In the near future, indexation may be achieved if we strictly follow a few rules. The journal has received support from Professor Antonio Spina-França Neto, editor-in-chief of *Arquivos de Neuro-Psiquiatria*, the most prestigious neurological journal in Brazil, and also from Abel Laerte Parker and other expert librarians of the Pan-American Health Organization and from the SCIELO (Scientific Library On-Line), a very successful Brazilian initiative designed for the on-line diffusion of Brazilian and Latin American scientific papers. If their guidance is followed, **Dementia&Neuropsychologia** will be on-line even before the end of its first year. Then, the first published papers will be retrospectively included in the on-line library and will be freely available to both national and international communities.When this landmark is accomplished, the journal will be attractive for the speed of on-line publication. The next challenge will be international indexation (PubMed, ISIS).

Papers will be published in English, but versions of tests, scales or special papers with features peculiar to Brazilian or Latin American spheres may be published in Portuguese or Spanish.

**Dementia & Neuropsychologia** is published quarterly and follows the *Uniform requirements for manuscripts submitted to biomedical journals*. The first issues are including reviews, original reports, short communications and case reports. In future issues, other sections will be included.

In this first issue it is necessary to also thank the Brazilian scientific community, the international members of the editorial board, and the advertisers for making possible to give this first step in a long, but hopefully very significant journey.

**Ricardo Nitrini** Editor-in-Chief

